# Lumbar vertebral osteoradionecrosis: a rare case report with 10-year follow-up and brief literature review

**DOI:** 10.1186/s12891-019-3024-z

**Published:** 2020-01-04

**Authors:** Cong Jin, Minghua Xie, Wengqing Liang, Yu Qian

**Affiliations:** Department of Orthopaedics, Shaoxing People’s Hospital (Shaoxing Hospital, Zhejiang University School of Medicine), Zhongxing North Road, Shaoxing, Zhejiang 312000 People’s Republic of China

**Keywords:** Lumbar vertebrae, Spine, Osteoradionecrosis, Rare case

## Abstract

**Background:**

Osteoradionecrosis (ORN) is a complication that occurs after radiotherapy for head or neck malignancies. ORN of the spine is rare, with only few cases affecting the cervical spine reported to date. To our knowledge, no case of lumbar ORN has been reported. We report a rare case of ORN in the lumbar spine that occurred 2 years after radiotherapy and perform a literature review.

**Case presentation:**

We present a case of lumbar ORN that occurred 2 years after radiotherapy for gallbladder carcinoma. The patient was successfully treated conservatively and followed up for > 10 years.

**Conclusions:**

ORN of the spine is a rare complication of radiotherapy. Spinal ORN is clinically described as a chronic disease with a slow onset. The most common presenting symptom of spinal ORN is pain. However, as ORN progresses, spinal kyphosis and instability can lead to neurological compression and thus to induced myelopathy or radiculopathy. Treatment of spinal ORN is comprehensive, including orthosis, medication, hyperbaric oxygen therapy, surgery, and new treatment combinations of pentoxifylline and tocopherol. The surgical rate for spinal ORN is relatively high.

## Background

Osteoradionecrosis (ORN) was defined as radiation-induced ischemic necrosis of bone and soft tissue in the absence of a local primary tumor, recurrence, or metastatic disease [1]. It was first reported by Regaud in 1922 and was thought to be a common complication that occurs after radiotherapy for head or neck malignancies [2]. The mandible was the most common site affected, with an incidence of 8–27%, followed by the maxilla, in 2–11% of patients [3]. In addition, it has also been documented in the cranium, pelvis, and sacrum [4]. ORN of the spine is rare, with only few cases affecting the cervical spine reported to date. To our knowledge, no case of lumbar ORN has been reported. We present a rare case of ORN in the lumbar spine 2 years after radiotherapy for gallbladder carcinoma with > 10 years’ follow-up.

## Case presentation

A 47-year-old woman was found to have an increased carbohydrate antigen 19–9 (CA19–9) level with no symptoms observed during a health checkup in June 2007. Positron emission computed tomography (PET) was suggested and revealed an obvious high uptake in the gallbladder. Gallbladder carcinoma was suspected. A radical cholecystectomy was performed in July 2007. The postoperative pathology was adenocarcinoma with invasion of the serosal layer, and no positive peripheral lymph nodes were detected. The tumor stage was T3 N0 M0. At 3 months after the operation, abdominal ultrasonography demonstrated retroperitoneal lymphadenopathy. Radiotherapy was administrated at a total dose of 3500 cGy in 14 fractions at the left gastric artery, pancreatic duodenum, and posterior region of the pancreatic head.

**Fig. 1:**
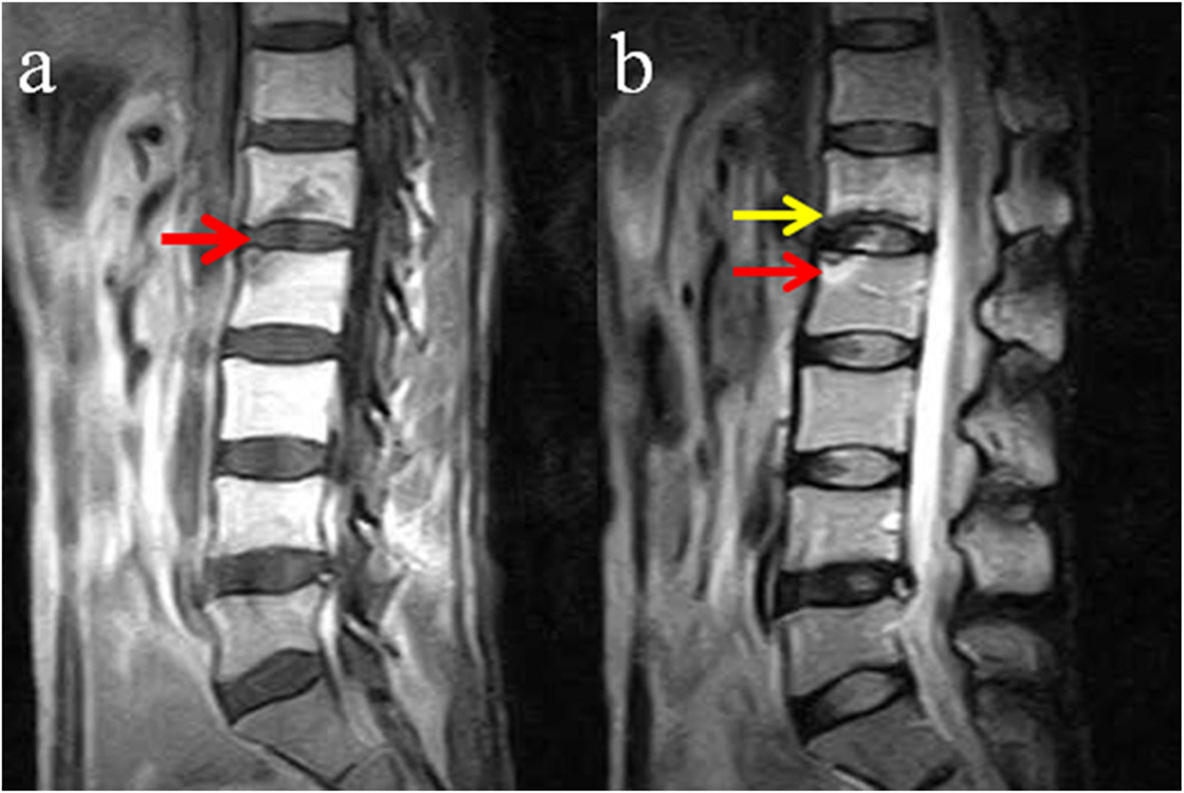
MRI images at 2 years after radiotherapy: **a** T1WI demonstrating a small hypointense area at the end plates of L1 and L2 (red arrow). **b** T2WI showing a hypointense signal change at the inferior end plate of L1 (yellow arrow) and high-low mixed signal change at the superior end plate of L2 (red arrow), without obvious signal changes of the intervertebral disk

The patient had an uneventful course thereafter until she visited our department 2 years after radiotherapy. She complained of a mild backache triggered by bending and twisting with no numbness or weakness of the lower limbs. She had no history of a recent trauma. Careful physical examination showed no tenderness on the lumbar spinous process. There was no decreased sensation of the lower limbs. Muscular strength and tendon reflexes of two lower limbs were normal. Magnetic resonance imaging (MRI) was scheduled. T1-weighted imaging (T1WI) revealed a small hypointense area at the end plate of L1 and L2 (Fig. 1a), whereas T2-weighted imaging (T2WI) revealed a hypointense signal change at the end plate of L1 and high-low mixed signal change at the end plate of L2 (Fig. 1b). The abnormal signal changes on MRI mimicked the Modic changes. A diagnosis of lumbar degenerative disease was the first consideration, and the patient was treated conservatively with medication (Celecoxib 200 mg, orally, twice daily and Eperisone 50 mg, orally, three times daily) and physical therapy.

**Fig. 2:**
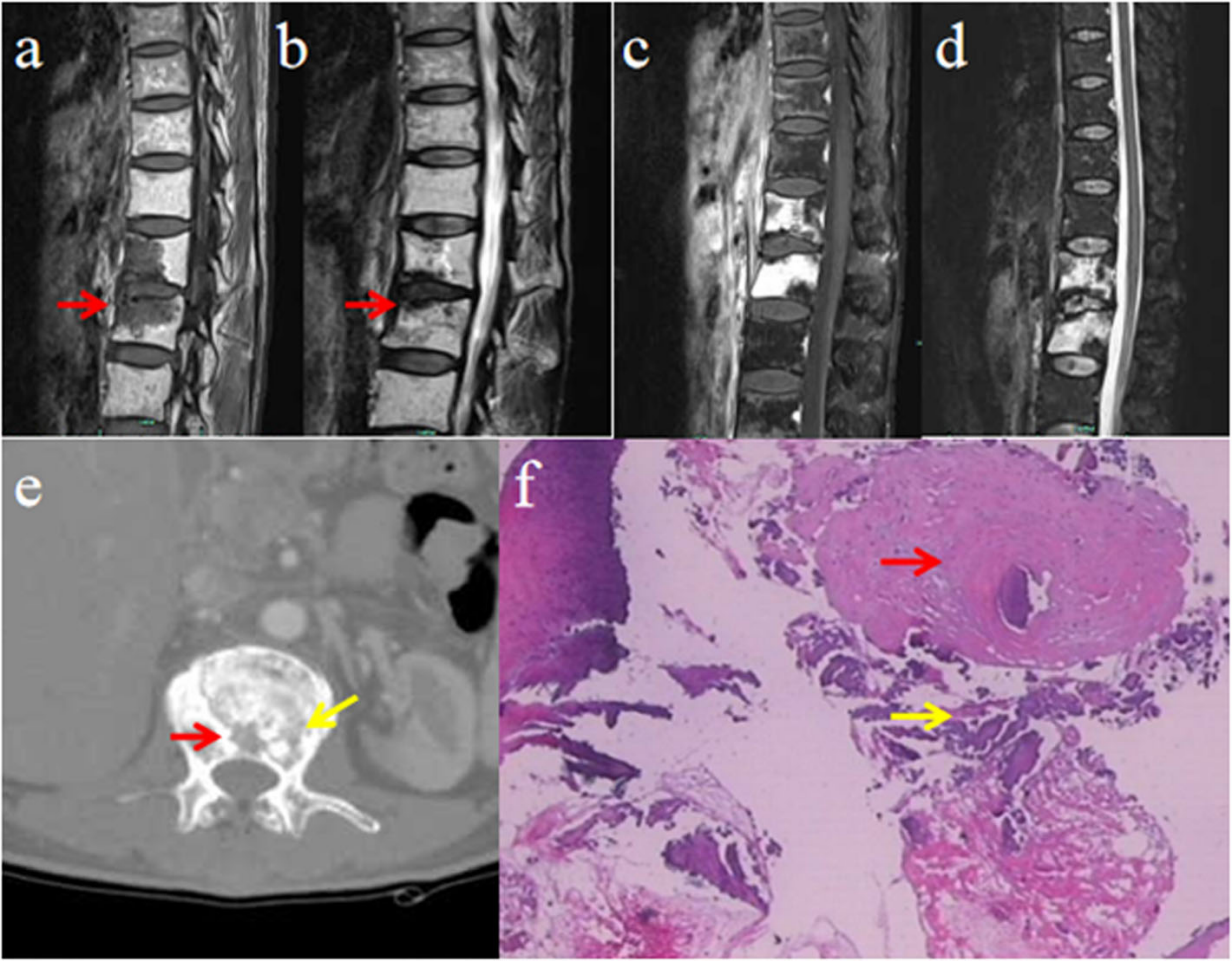
Radiographic data after conservative therapy for 6 months: **a** T1WI showing expansive hypointense signal changes (red arrow) in the L1 and L2 vertebral bodies. **b** T2WI demonstrating hypointense signal changes (red arrow) at the end plates of L1 and L2, with intervertebral disk involvement. **c**, **d** Enhanced signal changes in almost the entire L1 and L2 vertebral bodies on enhancing T1WI and T2WI. **e** CT scan showing osteolytic lesion (red arrow) with surrounding sclerotic bone (yellow arrow) in the vertebral body without expansion to the vertebral pedicle or laminae. **f** Histological examination result showing fragments of bone (red arrow) and fibrous connective tissues with focal necrosis (yellow arrow) (hematoxylin and eosin staining; × 20 magnification)

Unfortunately, the patient complained of persistent backache that was aggravated after conservative therapy for 6 months. Physical examination revealed reduced lumbar motion and tenderness at the spinous process of L1 and L2. The sensation, muscular strength and tendon reflexes of two lower limbs were normal. Laboratory investigations including white blood cell counts, fraction of neutrophils, C-reactive protein, erythrocyte sedimentation rate and tumor markers were normal. MRI revealed expansive hypointense signal changes with enhancement on T1WI and T2WI at the vertebral body of L1 and L2 (Fig. 2a–d). In addition, computed tomography (CT) revealed an osteolytic lesion with surrounding sclerotic bone in the vertebral body without expansion to the vertebral pedicle or laminae (Fig. 2e). Radionuclide bone imaging indicated a radioactivity concentration on L1 and L2. Thus, the impression of primary or metastatic tumor cannot be excluded. A CT-guided biopsy of L1 and L2 vertebral bodies was applied, and histological examination revealed fragments of bone and fibrous connective tissues with focal necrosis (Fig. 2f). Definitely, no evidence of metastatic carcinoma was found. We concluded that the definite diagnosis was lumbar spine ORN secondary to the radiotherapy 2 years earlier. The patient received conservative treatment including medication (Celecoxib 200 mg, orally, twice daily and calcium carbonate 600 mg, orally, daily) and orthosis. The patient was followed up every 3 months. Her back pain was completely relieved after conservative treatment for 6 months.

**Fig. 3:**
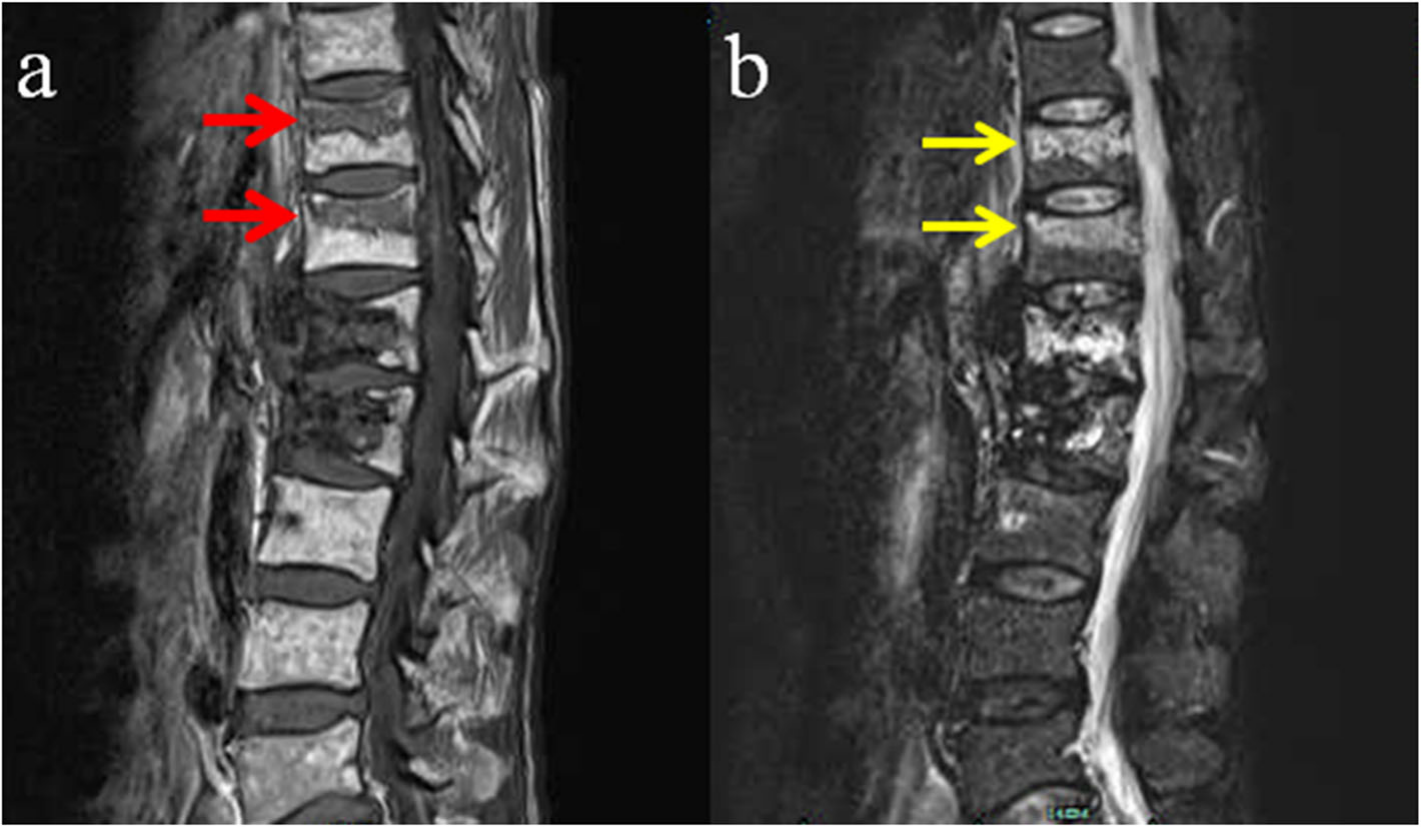
MRI images at 9 years after radiotherapy following trauma: **a** T1WI demonstrating hypointense signal changes of the T11 and T12 vertebrae (red arrow). **b** T2WI showing hyperintense signal changes of the T11 and T12 vertebrae (yellow arrow)

**Fig. 4:**
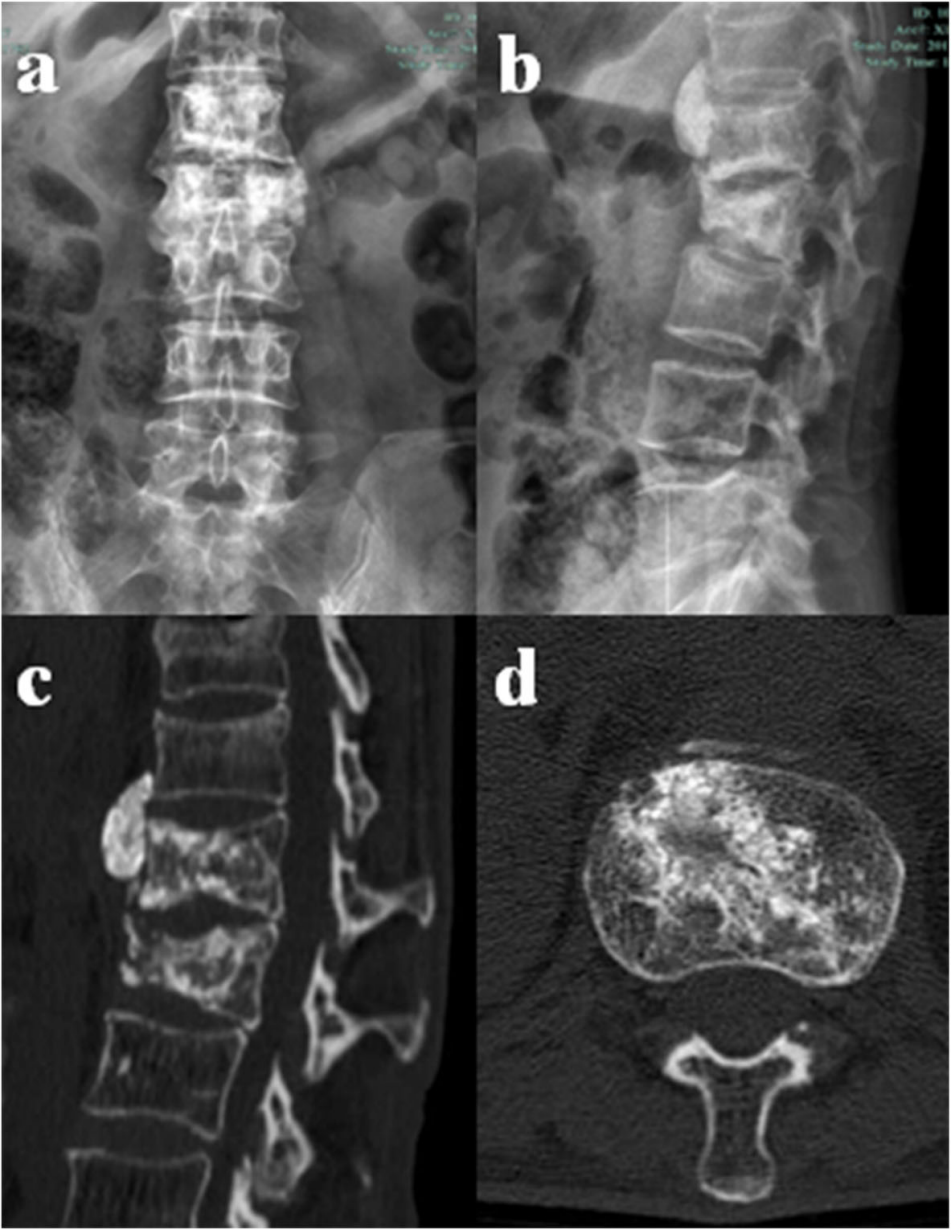
Radiographic data at 10 years after radiotherapy: **a**, **b** Radiograph showing sclerotic bone in the L1 and L2 vertebral body. The L2 vertebral body was compressed, which induced local kyphosis of the thoracolumbar spine. **c**, **d** CT scan demonstrating calcification or sclerotic bone without obvious osteolytic lesion in the L1 and L2 vertebral bodies

At 9 years after radiotherapy, the patient fall from 3 m-high balcony, and visited our emergency department. She complained of back pain, and physical examination revealed tenderness at the spinous processes of T11 and T12. MRI revealed hypointense signal changes of T11 and T12 vertebrae on T1-weighted imaging (Fig. 3a) and hyperintense signal changes on T2-weighted imaging (Fig. 3b). Compression fractures of T11 and T12 were diagnosed, and conservative treatment including bedrest for 6 weeks and medication (calcitrol 0.25 μg, orally, daily and calcium carbonate 600 mg, orally, daily) was applied. At the last follow-up (10 years after radiotherapy), no backache or other symptoms were observed. Mild thoracolumbar kyphosis was observed on physical examination. Radiographic images showed sclerotic bone in the L1 and L2 vertebral bodies and compression of the L2 vertebral body that induced a mild thoracolumbar kyphosis (Fig. 4a, b). CT scanning demonstrated calcification or sclerotic bone with no obvious osteolytic lesion in the L1 and L2 vertebral bodies (Fig. 4d, e).

## Discussion and conclusion

Radiotherapy is applied in various tumors, either as a primary or adjuvant treatment with surgery or chemotherapy. Radiation may cause damages to the skeletal system, depending on the age and health of the patient, size and location of the radiation field, and dose and fractionation of the beam [5]. Doses > 4000 cGy have been reported to be usually required to cause this irreversible bone injury, and the rate of necrosis was up to 50% with dose of > 7500 cGy [5]. Previous cervical ORN cases have been reported with a cumulative dose of 3000–6000 cGy [4]. Our patient received a total of 3500 cGy of focused radiotherapy and 14 doses at 250 cGy for the treatment of T3 N0 M0 gallbladder carcinoma, which is in the line with previously published reports.

During the past century, several theories have been proposed about the pathogenesis of ORN. The pathophysiology of ORN was believed to be a radiation-induced metabolic and homeostatic deficiency. In 1983, Marx proposed the “three H” hypothesis to describe post-radiation injury, which has been the most widely accepted hypothesis [6]. It is characterized by the formation of hypoxic, hypovascular, and hypocellular tissues. It was hypothesized that radiation-induced injury caused bone to be unable to increase its metabolic and nutritional requirements and thus to replace the normal collagen and cellular components lost through routine wear. This results in tissue breakdown and necrosis. In addition, in 2004, a new theory for the pathogenesis of ORN was reported [7] and revealed that damage to bone is caused by radiation-induced fibrosis. Cells in bone are damaged as a result of acute inflammation, free radicals, and chronic activation of fibroblasts by a series of growth factors. The destruction of cells, accompanied by vascular thrombosis, leads to local ischemia, necrosis of micro-vessels, and tissue breakdown.

ORN is clinically described as a chronic disorder with a slow onset. It begins with an acute phase response to radiation that consists of bone marrow edema and mild surrounding soft tissue inflammation. ORN gradually progresses to the chronic phase, which includes vascular damage that leads to a combination of osteitis and discitis. ORN of the jaw has been reported to commonly present within 2 years after radiation treatment but has been described to present up to 20 to 30 years after the initial radiotherapy [8]. In addition, we reviewed that the mean time to onset of previous cervical ORN cases was 112 months, and the longest time was even up to 42 years [4, 7–12]. The onset time of spinal ORN seemed even longer than that of ORN of the mandible or maxilla. In this case, the occurrence time of lumbar ORN was around 2 years after radiotherapy. Compared with previous spinal ORN cases, the present case seemed to have a relatively earlier onset time.

The classification of ORN proposed by Marx is widely acceptable [13]. He describes three types of ORN. Type 1 ORN develops shortly after radiation treatment as the synergistic effect of surgical trauma and radiation injury. Type 2 ORN occurs within a few years after the radiation due to progression of endarteritis and vascular occlusion. This type of ORN always follows a traumatic event and rarely occurs before 2 years after treatment, commonly occurring after 6 years. Type 3 ORN occurs spontaneously without traumatic events between 6 months and 3 years after radiotherapy. This type is associated with immediate cellular damage and death due to radiotherapy. In this case, the patient had ORN 2 years after radiotherapy without a traumatic factor, so the classification of ORN was type 3 according this classification system. In the previous cervical ORN cases reported, type 3 ORN occurred most frequently (29/31, 93.5%), followed by type 1 ORN (2/31, 6.5%) [4, 7–12].

The most common presenting symptom of ORN is pain. As shown in this case, the only symptom present was chronic back pain. Khorsandi reported that all patients with cervical spine ORN presented with neck pain [12]. However, as ORN progresses, spinal kyphosis and instability can also lead to neurological compression and induced myelopathy or radiculopathy. In our review, most patients (18/31, 58%) with cervical ORN presented with symptoms of neurological injury at the end stage [4, 7–12]. Nevertheless, the common symptom of back pain is often overlooked as numerous etiological factors influenced. Patients with spinal ORN do not have any severe neurological symptoms and can be treated conservatively most of the time, as they mainly present pain, for which analgesics are adequate. This is the main reason why most patients with spinal ORN are not identified until complications occur.

Given the history of gallbladder carcinoma in our patient and the site of the lesion at the lumbar spine, differentiation from recurrent or metastatic disease was essential, as the throacolumbar spine is the leading site of spinal metastasis, and the diseases require very different management strategies. Usually, no special signs of ORN are observed on radiography, but dynamic radiography also plays an important role to demonstrate spinal kyphosis and instability caused by the lesion of the spinal ORN [12]. In this study, radiography or dynamic radiography was not conducted before the MRI arranged. It is a limitation of this case report. MRI, CT, and PET scan have been demonstrated to be useful imaging tools for the diagnosis of ORN but have also revealed limitations in distinguishing metastatic or recurrent lesions from radiation-induced injury. The hypointense areas on T1WI and T2WI that corresponded to the intervertebral vacuum cleft sign suggested ORN [5]. Mut proposed that MRI of ORN demonstrates T1 hypointensity with variable T2 signal [14]. The signal changes in the intervertebral disk on MRI were useful for the differential diagnosis of ORN from metastatic disease, as the intervertebral disk was mostly not involved in spinal tumor. Khorsandi reported that the intervening disk space had a hypointense signal on T1WI and T2WI in three of four cases [11], while Wu et al. reported that the hypointense signal on T1WI and hyperintense signal on T2WI of the intervertebral disk indicated discitis, an important signal of end-stage spinal ORN [5]. In our case, the signal changes on MRI were variational as the progress of spinal ORN. Thus, further study on MRI findings of spinal ORN is essential for understanding the natural history of spinal ORN. Alhilali also suggest that besides MRI findings, bony sclerosis is a useful CT imaging feature for differentiating ORN from recurrent tumors [15]. In addition, PET scan has been shown to be a promising method for discriminating between radiation-induced change and tumor recurrence. Dholam reported that differential diagnosis between ORN and bony metastasis may be possible with low FDG uptake of ORN on PET scan [16]. However, the inflammatory response associated with ORN may be interpreted as a false-positive strong uptake, and false-positive PET scan results have been reported in post-radiotherapy patients [16]. The specificity of the examination in distinguishing the etiology is approximately 85% [17]. Utility of single photon emission computed tomography (SPECT) using various radio-pharmaceuticals has been reported to diagnose ORN and differentiate it from metastasis. Deshpande found that TI-201 SPECT clarified 75% (three of the four) of the false-positive PET to be ORN. Thus, SPECT may be used to reduce false-positive rates and improve specificity [18]. Owing to the lack of specificity of imaging studies in the differential diagnosis from recurrent tumors, bone biopsies may be invasive in special cases but can be useful for making a definite diagnosis of ORN.

Besides differentiation from metastatic disease, differential diagnosis from pyogenic spondylitis or spinal tuberculosis was necessary. Firstly, patients with pyogenic spondylitis or spinal tuberculosis were more frequently associated with constitutional symptoms such as fever, shivers and loss of body weight. Secondly, Kim found that pyogenic spondylitis was commonly associated with high white blood cell counts, high fraction of neutrophils, elevated C-reactive protein and erythrocyte sedimentation rate [19], whereas they may be normal or slightly elevated in ORN. In addition, paravertebral abscess was a specific imaging feature for pyogenic spondylitis or spinal tuberculosis on CT or MRI, however, in ORN, less paraspinal soft tissue was involved.

Treatment of spinal ORN is comprehensive, and strategies have included orthosis, medication (anti-inflammatory analgesic, clodronate, and corticosteroids), hyperbaric oxygen therapy, surgery, and new treatment combinations of pentoxifylline and tocopherol. Orthosis is essential for patients with spinal ORN to support the physical loading of the spinal column, relieve pain, and prevent spinal deformity. The most common symptom of patients with spinal ORN was back pain, and painkillers such as non-steroidal anti-inflammatory drugs are necessary. Some reports have recommended clodronate for ORN owing to its effect on osteoclast inhibition, but evidence supporting its usefulness is insufficient [6]. In addition, hyperbaric oxygen therapy was reported to be an excellent adjunct treatment for ORN, as it stimulated angiogenesis, fibroblast, and collagen formation [20]. Khorsandi also reported that hyperbaric oxygen therapy is associated with significantly improved clinical and radiographic outcomes for a patient with cervical ORN [11]. Surgery for spinal ORN is indicated when kyphosis or instability is progressive and leads to spinal cord compression. The surgical principles for spinal ORN are debridement of necrotic tissue, decompression of neural structures, and reestablishment of spinal alignment and stability using a structural graft [4]. Anterior spinal surgery was also recommended owing to its advantages of excellent removal of necrotic bone, direct spinal cord decompression, and good fusion bed preparation in an irradiated surgical area. In the previous cervical ORN cases reported, the surgical rate for ORN was 52% (16/31), indicating that most patients with spinal ORN cannot be cured with conservative treatment [4, 7–12]. In our case, the patient was successfully treated conservatively. This implied that ORN of the cervical spine may easily cause neurological complications as compared with that of the lumbar spine. Moreover, the pathogenesis of ORN is caused by radiation-induced fibrosis, as proposed in the new theory mentioned earlier. New therapies have been developed that include pentoxifylline, a vasodilator that inhibits fibrosis, and tocopherol (vitamin E) to reduce the damage caused by free radicals [7].

In this case, an interesting finding was the adjacent vertebral fractures that occurred after ORN was cured following trauma. During the healing of ORN, bony sclerosis may cause increased strength and decreased elasticity of the vertebrae. It may increase the risk of adjacent vertebral fracture, similarly to the biomechanical mechanism of the adjacent vertebral fracture after percutaneous kyphoplasty.

In summary, ORN of the spine is a rare complication of radiotherapy. We present a case of lumbar ORN after radiotherapy for gallbladder carcinoma, which was successfully treated conservatively. ORN is described clinically as a chronic disease with a slow onset. The most common presenting symptom of ORN is pain. However, as ORN progresses, spinal kyphosis and instability lead to neurological compression, and induced myelopathy or radiculopathy. Treatment of spinal ORN is comprehensive, and the surgical rate for spinal ORN was relatively high, indicating that most patients with spinal ORN cannot be cured with conservative treatment.

## Data Availability

The authors declare that all data supporting the findings of this study are available within the article.

## Abbreviations

CA19–9: Carbohydrate antigen 19–9
CT: Computed tomography
MRI: Magnetic resonance imaging
ORN: Osteoradionecrosis
PET: Positron emission computed tomography
SPECT: Single photon emission computed tomography
T1WI: T1-weighted imaging
T2WI: T2-weighted imaging

## References

[1] Meixel AJ, Hauswald H, Delorme S, Jobke B (2018). From radiation osteitis to osteoradionecrosis: incidence and MR morphology of radiation-induced sacral pathologies following pelvic radiotherapy. Eur Radiol.

[2] Huang WB, Wong STS, Chan JYW (2018). Role of surgery in the treatment of osteoradionecrosis and its complications after radiotherapy for nasopharyngeal carcinoma. Head Neck.

[3] Kuhnt T, Stang A, Wienke A, Vordermark D, Schweyen R, Hey J (2016). Potential risk factors for jaw osteoradionecrosis after radiotherapy for head and neck cancer. Radiat Oncol.

[4] Powell DK, Jacobson AS, Kuflik PL, Persky MS, Silberzweig JE, Khorsandi AS (2013). Fibular flap reconstruction of the cervical spine for repair of osteoradionecrosis. Spine J.

[5] Wu LA, Liu HM, Wang CW, Chen YF, Hong RL, Ko JY (2012). Osteoradionecrosis of the upper cervical spine after radiation therapy for head and neck cancer: differentiation from recurrent or metastatic disease with MR imaging. Radiology.

[6] Rivero JA, Shamji O, Kolokythas A (2017). Osteoradionecrosis: a review of pathophysiology, prevention and pharmacologic management using pentoxifylline, alpha-tocopherol, and clodronate. Oral Surg Oral Med Oral Pathol Oral Radiol.

[7] Fan H, Kim SM, Cho YJ, Eo MY, Lee SK, Woo KM (2014). New approach for the treatment of osteoradionecrosis with pentoxifylline and tocopherol. Biomater Res.

[8] Yao CY, Zhou GR, Wang LJ, Xu JH, Ye JJ, Zhang LF, He X, Chen ZZ, Huang SF (2018). A retrospective dosimetry study of intensity-modulated radiotherapy for nasopharyngeal carcinoma: radiation-induced brainstem injury and dose-volume analysis. Radiat Oncol.

[9] Raza A, Islam M, Lakshmanan P. Secondary atlanto-odontoid osteoarthritis with osteoradionecrosis of upper cervical spine mimicking metastasis. BMJ Case Rep. 2013;2013. 10.1136/bcr-2013-009319 PMC3794319 24038287

[10] Rashid MZ, Ariffin MH, Rhani SA, Baharudin A, Ibrahim K (2017). Osteoradionecrosis in subaxial cervical spine - a rare and devastating complication: a case report. Malays Orthop J.

[11] Khorsandi AS, Su HK, Mourad WF, Urken ML, Persky MS, Lazarus CL, Jacobson AS (2015). Osteoradionecrosis of the subaxial cervical spine following treatment for head and neck carcinomas. Br J Radiol.

[12] Cheung JP, Wei WI, Luk KD (2013). Cervical spine complications after treatment of nasopharyngeal carcinoma. Eur Spine J.

[13] He Y, Liu Z, Tian Z, Dai T, Qiu W, Zhang Z (2015). Retrospective analysis of osteoradionecrosis of the mandible: proposing a novel clinical classification and staging system. Int J Oral Maxillofac Surg.

[14] Mut M, Schiff D, Miller B, Shaffrey M, Larner J, Shaffrey C (2005). Osteoradionecrosis mimicking metastatic epidural spinal cord compression. Neurology.

[15] Alhilali L, Reynolds AR, Fakhran S (2014). Osteoradionecrosis after radiation therapy for head and neck cancer: differentiation from recurrent disease with CT and PET/CT imaging. AJNR Am J Neuroradiol.

[16] Dholam KP, Singh GP, Agarwal JP, Mahajan A (2019). 18F FDG uptake due to late-onset Osteoradionecrosis for Tongue Base carcinoma. Clin Nucl Med.

[17] Wang CH, Liang JA, Ding HJ, Yang SN, Yen KY, Sun SS, Kao CH (2010). Utility of TL-201 SPECT in clarifying false-positive FDG-PET findings due to osteoradionecrosis in head and neck cancer. Head Neck.

[18] Deshpande SS, Thakur MH, Dholam K, Mahajan A, Arya S, Juvekar S (2015). Osteoradionecrosis of the mandible: through a radiologist's eyes. Clin Radiol.

[19] Kim CJ, Song KH, Jeon JH, Park WB, Park SW, Kim HB, Oh MD, Choe KW, Kim NJ (2010). A comparative study of pyogenic and tuberculous spondylodiscitis. Spine (Phila Pa 1976).

[20] Sultan A, Hanna GJ, Margalit DN, Chau N, Goguen LA, Marty FM, Rabinowits G, Schoenfeld JD, Sonis ST, Thomas T (2017). The use of hyperbaric oxygen for the prevention and Management of Osteoradionecrosis of the jaw: a Dana-Farber/Brigham and Women's Cancer center multidisciplinary guideline. Oncologist.

